# The BOLD response in primary motor cortex and supplementary motor area during kinesthetic motor imagery based graded fMRI neurofeedback

**DOI:** 10.1016/j.neuroimage.2018.09.007

**Published:** 2019-01-01

**Authors:** David M.A. Mehler, Angharad N. Williams, Florian Krause, Michael Lührs, Richard G. Wise, Duncan L. Turner, David E.J. Linden, Joseph R. Whittaker

**Affiliations:** a MRC Centre for Neuropsychiatric Genetics and Genomics, Division of Psychological Medicine and Clinical Neurosciences, School of Medicine, Cardiff University, Cardiff, CF24 4HQ, United Kingdom; b Cardiff University Brain Research Imaging Centre, School of Psychology, Cardiff University, Cardiff, CF24 4HQ, United Kingdom; c Donders Institute for Brain, Cognition and Behaviour Radboud University Medical Center, 6500 HB, Nijmegen, The Netherlands; d Faculty of Psychology and Neuroscience, Maastricht University, Universiteitssingel 40, 6229 ER, Maastricht, The Netherlands; e Brain Innovation B.V, Oxfordlaan 55, 6229 EV, Maastricht, The Netherlands; f Neurorehabilitation Unit, School of Health, Sport and Bioscience, University of East London, London, E15 4LZ, United Kingdom; g School of Mental Health and Neuroscience, Faculty of Health, Medicine and Life Sciences, Maastricht University, Universiteitssingel 40, 6229 ER, Maastricht, The Netherlands; h School of Physics and Astronomy, Cardiff University, Cardiff, CF24 3AA, United Kingdom

## Abstract

There is increasing interest in exploring the use of functional MRI neurofeedback (fMRI-NF) as a therapeutic technique for a range of neurological conditions such as stroke and Parkinson's disease (PD). One main therapeutic potential of fMRI-NF is to enhance volitional control of damaged or dysfunctional neural nodes and networks via a closed-loop feedback model using mental imagery as the catalyst of self-regulation. The choice of target node/network and direction of regulation (increase or decrease activity) are central design considerations in fMRI-NF studies. Whilst it remains unclear whether the primary motor cortex (M1) can be activated during motor imagery, the supplementary motor area (SMA) has been robustly activated during motor imagery. Such differences in the regulation potential between primary and supplementary motor cortex are important because these areas can be differentially affected by a stroke or PD, and the choice of fMRI-NF target and grade of self-regulation of activity likely have substantial influence on the clinical effects and cost effectiveness of NF-based interventions. In this study we therefore investigated firstly whether healthy subjects would be able to achieve self-regulation of the hand-representation areas of M1 and the SMA using fMRI-NF training. There was a significant decrease in M1 neural activity during fMRI-NF, whereas SMA neural activity was increased, albeit not with the predicated graded effect. This study has important implications for fMRI-NF protocols that employ motor imagery to modulate activity in specific target regions of the brain and to determine how they may be tailored for neurorehabilitation.

## Highlights

• Graded fMRI neurofeedback can separate self-regulation effects from general cognitive ones.
• There is a significant negative BOLD response in M1 during kinaesthetic motor imagery.
• SMA shows robust positive BOLD responses during kinaesthetic motor imagery.
• Participants can target discrete levels of BOLD response in SMA using kinaesthetic motor imagery.
• For our paradigm neurofeedback provides only minimal extra control of BOLD signals in SMA.

## 1 Introduction

Real-time functional magnetic resonance imaging neurofeedback (fMRI-NF) is currently being explored as a non-invasive technique to improve motor rehabilitation outcome in neurological conditions including Parkinson's disease (PD) (Subramanian et al., 2011) and stroke (Liew et al., 2016; Linden and Turner, 2016; Wang et al., 2017). Recently, the first randomized controlled trial for fMRI-NF in PD suggested that the technique may lead to clinically significant motor improvements when combined with physical exercise (Subramanian et al., 2016). Initial clinical findings and theoretical considerations suggest that self-regulation training with neurofeedback may particularly benefit movement initiation and fluidity (Linden and Turner, 2016). Regarding stroke, a recent systematic review concluded that fMRI-NF training can lead to learned modulation of brain signals and may be associated with beneficial behavioral changes (Mihara and Miyai, 2016; Wang et al., 2017). Hence, there is an increased interest in improving fMRI-NF protocols to maximize the potential of the associated clinical outcomes (Stoeckel et al., 2014).

One central consideration when designing fMRI-NF interventions is the choice of suitable target brain region(s) from which feedback is provided during training. Although some techniques work with implicit feedback that does not require cognitive strategies (Watanabe et al., 2017), most fMRI-NF training protocols currently employ mental strategies such as visual imagery (Habes et al., 2016), affective imagery (Johnston et al., 2011; Linden et al., 2012; Young et al., 2017) or motor imagery tasks (Auer et al., 2016; Berman et al., 2012; Blefari et al., 2015; Chiew et al., 2012; Subramanian et al., 2011, 2016). Therefore, target regions are often selected based on their anticipated involvement in the respective mental imagery process.

For fMRI-NF of motor areas, most protocols instruct participants to perform motor imagery (Blefari et al., 2015; Sepulveda et al., 2016; Sitaram et al., 2012; Subramanian et al., 2016). FMRI correlates of motor imagery have been well documented in healthy individuals (Guillot et al., 2009; Jeannerod, 2001; Munzert et al., 2009; Sharma and Baron, 2013; Solodkin et al., 2004; Vingerhoets et al., 2002) and preliminary data from stroke survivors also showed robust neural correlates of motor imagery (Bajaj et al., 2015a; b; Kraft et al., 2015; Sharma et al., 2009; Wong et al., 2013) in the ventral premotor cortex (vPMC), dorsal premotor cortex (dPMC), and the supplementary motor area (SMA (Hetu et al., 2013);) This finding has been corroborated by recent motor imagery based fMRI-NF studies (Blefari et al., 2015; Sepulveda et al., 2016; Subramanian et al., 2016). Further, neuroimaging studies of motor imagery and motor execution indicate that both tasks share overlapping motor networks, although differences have been identified (Hanakawa, 2016)(for a review see (Hetu et al., 2013)). In particular for the primary motor cortex (M1), the motor imagery literature consists of mixed findings (Hetu et al., 2013), and it is not clear whether M1 can be robustly activated during motor imagery training. It thus remains an open question whether M1 represents an effective target region for motor imagery-based fMRI-NF paradigms that entrain upregulation.

The first aim of this study was thus to compare self-regulation abilities for M1 and a higher motor region (SMA) in the same participants. Its second aim was to explore the feasibility of graded neurofeedback of motor regions. The rationale for graded neurofeedback protocols, where participants are not just trained to upregulate activity in the target region but to upregulate it to different specified levels, is that they provide more degrees of freedom for adaptive neurorehabilitation programmes and for neural communication in a BCI framework. ‘Gradual’ fMRI-NF has been introduced recently (Sorger et al., 2016) (referred to as ‘graded’ in this study). It offers increased scope for evaluating training success, for example by assessing how well participants can attain discrete magnitudes of BOLD signal changes through supervised mental imagery. Given that it is well established that certain motor regions, including the SMA, are activated during non-supervised motor imagery, significant BOLD activation of a motor region during supervised motor imagery training (e.g. fMRI-NF training) does not by itself provide sufficient evidence of volitional control success. Graded fMRI-NF training, in contrast, allows one to gauge the degree of control gained over the activation level of a target region.

In this study, we aim to lay the foundation for the further development of motor-imagery based neurofeedback of areas in the motor network. The graded NF protocol separates the general effects of motor imagery from the more specific neurofeedback targeted effect of volitional self-control as reflected in the BOLD signal. Participants were required to target two discrete levels (*low* and *high* level), while feedback was provided from either the SMA or M1. The novelty of our design is that it allows this to be assessed, alongside the typical neurofeedback effect, within a single factorial framework by contrasting which of the two regions feedback information is derived from. The purpose of this study is to provide the foundation for future development of fMRI-NF protocols for neurorehabilitation, in particular stroke and PD. Given that the majority of published studies have targeted cortical motor regions (Wang et al., 2017), typically premotor areas or primary motor areas, our aim was to determine which region is most suitable for motor imagery based NF. Our graded NF design allows us to address two important questions;

1) When combined with a kinesthetic motor imagery strategy, are SMA and M1 robustly activated? This is a key prerequisite for further NF upregulation training.
2) Do subjects show better separation between discrete target levels for a particular motor region when provided with feedback from that region?

We hypothesized that SMA based fMRI-NF training would show robust activation in the SMA, but no activation in M1. Given previous unsuccessful attempts of M1 fMRI-NF training (Berman et al., 2012; Blefari et al., 2015; Chiew et al., 2012), we hypothesized that M1 based fMRI-NF training would not yield M1 activation, whilst the SMA as a region involved in motor imagery would still show activation. Lastly, we expected to find differences in SMA activation between target levels when feedback was provided from the SMA (*active* condition), but not when feedback was provided from M1 (*passive* condition).

## 2 Methods

### 2.1 Experiment

#### 2.1.1 Participants

Twenty healthy participants were recruited from an internal experiment database. All participants gave written informed consent and the Cardiff University School of Psychology Ethics Committee approved the study. Data from three participants were excluded due to technical difficulties with the feedback system during scanning. Data from the remaining 17 participants (8 female; age 26.6 ± 5.5 [Mean ± SD] years) were included in the data analysis.

#### 2.1.2 Motor imagery and post-training questionnaires

Motor imagery can be performed in two different modalities. It can be mainly visual or mainly kinesthetic. Visual motor imagery focuses primarily on visual mental imagery (either from a first or a third person perspective), whereas kinesthetic motor imagery usually involves taking a first-person perspective and imagining the feeling and experience of movements without overt movement (Hanakawa, 2016). Previous literature suggests that only a minority (27%) of studies had specified the modality that participants used during motor imagery training (Hetu et al., 2013), indicating that participants received inconsistent or unspecific instructions between or across studies. Kinesthetic and visual motor imagery are associated with distinct neural activity patterns (Guillot et al., 2009; Kilintari et al., 2016; Solodkin et al., 2004) and this may partly explain the inconsistent findings reported for M1in the motor imagery literature. In the present study participants completed a standard motor imagery questionnaire before the scanning session, which included self-ratings for both visual and kinesthetic motor imagery tasks on a scale from 1 to 7 (Gregg et al., 2010). The questionnaire was used (1) to make participants more aware of the distinction between the two different motor imagery forms and (2) to assess their self-rated baseline ability of motor imagery. After scan sessions, participants were asked to fill in a questionnaire about their experience.

#### 2.1.3 Instructions for localizer and neurofeedback training

Among the studies that have reported the modality of motor imagery instructions, and reported M1 activation, only kinesthetic motor imagery activates M1 (Hetu et al., 2013; Solodkin et al., 2004; Stinear et al., 2006) and therefore for this study participants used only kinesthetic motor imagery strategies (Guillot et al., 2009). Specifically, participants were instructed to imagine the body sensation and experience of moving both of their hands, without actually moving them, and while remaining relaxed. Participants could chose the precise movement they imagined, but were told to imagine a movement that they regularly perform (e.g. playing an instrument, doing housework or typing).

During the localizer run, participants were presented with an empty thermometer on the screen. They were instructed to perform a paced bimanual motor execution task (finger opposition) during task periods (as indicated by green arrows). Specifically, numbers from 1 to 4 were presented at a frequency of 1.33 Hz to pace the movement during task periods. During rest periods (as indicated by red arrows) participants were instructed to rest and relax. Participants were reminded that no feedback was presented during the localizer run.

For the neurofeedback runs, it was explained to participants that bars in the thermometer represented the activity level in the target region. Participants were instructed that their goal was to use kinesethetic motor imagery to control the feedback by filling up the bars of the thermometer display. They were further instructed to attempt to maintain the activation at target levels by adjusting their motor imagery strategy (e.g. by changing the speed and/or intensity of the imagined movement). Besides these aspects, participants were not restricted in the content of motor imagery and were allowed to imagine any activity or sport that involved movements of both hands. Indeed, participants were encouraged to explore which motor imagery strategies were most effective in gaining control over the filling bars of the thermometer display. Furthermore, participants were instructed that mental imagery strategy changes would not immidiately effect the feedback because of the hemodynamic delay. Between scans they were also reminded to remain still, relaxed and avoid muscle contractions during scanning.

#### 2.1.4 Post-training questionnaire

After scan sessions participants completed a questionnaire about their experience, which was designed to rate their perception of the following aspects of the experiment: (1) controllability of feedback for both ROIs, (2) the difficulty of filling up bars in the thermometer, and (3) the difficulty in maintaining the thermometer at a discrete level. For question 1, a score of 10 indicated high controllability, whereas for question (2) and (3), a score indicated high difficulty. Question (2) and (3) were added as exploratory items to the questionnaire after data collection had already started and were thus only recorded in 10 participants.

#### 2.1.5 MRI data acquisition

Imaging data were acquired using on 3T General Electric HDx with an eight-channel receiver head coil. Blood oxygenation level-dependent (BOLD) fMRI runs (see description below) were measured with a T_2_^∗^-weighted gradient-echo echo-planar imaging (EPI) sequence. Each functional EPI volume contained 25 slices of 2.5-mm thickness, with 0.5-mm inter-slice spacing (in-plane resolution = 3 mm, matrix size = 64 × 64, FoV (field of view) = 192 mm, TR (repetition time) = 1550 ms, TE (echo time) = 30 ms, flip angle = 80°, orientation = transversal). High-resolution structural images were acquired before the first functional scan using a fast-spoiled gradient echo (FSPGR) sequence with 172 contiguous sagittal slices of 1-mm thickness (voxel size: 1 × 1 × 1 mm, TR = 7.9 s, TE = 3.0 ms, flip angle = 20°, FoV = 256 × 256 × 172 mm).

#### 2.1.6 Monitoring of physiological confounds

A recent critical systematic review of fMRI-NF (Thibault et al., 2018) has highlighted the importance of monitoring subject physiology during scans, to determine if BOLD signal changes are significantly confounded by physiological noise (Murphy et al., 2013). Therefore, we recorded pulse waveforms using pulse oximetry, from which subject heart-rate (HR) time series were calculated. We also recorded the partial pressure of end-tidal carbon dioxide (P_ET_ CO_2_) using a nasal canula for the majority of participants. Data was recorded with Spike2 (version 5.21, Cambridge Electronics Design Limited, Cambridge, UK) for 10 (P_ET_ CO_2_) and 13 (pulse traces) participants, respectively.

#### 2.1.7 Real-time fMRI neurofeedback setup

Reconstructed DICOM images were transmitted in real-time from the MR computer to a dedicated analysis computer. Turbo-BrainVoyager (TBV) software (BrainInnovation B.V., Maastricht, The Netherlands, version 3.2) was used for real-time online pre-processing and analysis of BOLD signals including motion correction (with respect to the first volume of the functional localizer) and spatial smoothing (4 mm full width at half maximum; FWHM).

#### 2.1.8 Scan session

**Fig. 1:**
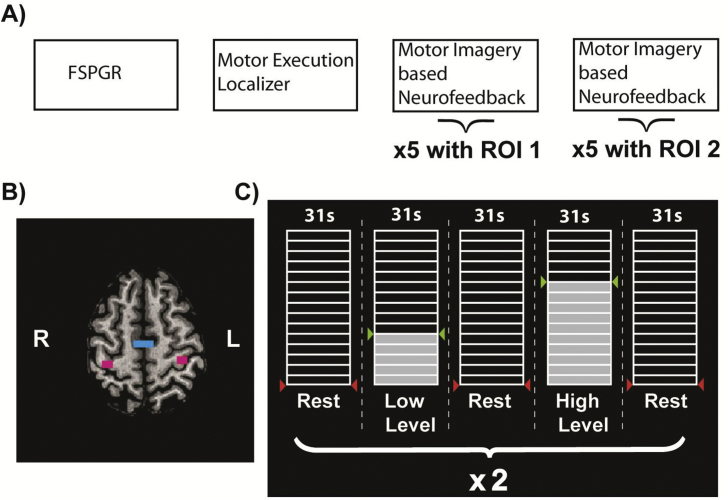
Experimental Setup. A) show sequence of scans during train session, b) shows target region with SMA in blue and M1 in magenta for an exemplary participant. C) shows an exemplary sequence of the thermometer display with *low* level and *high* level training blocks, flanked by rest blocks.

The sequence of scans is shown in Fig. 1A. Subjects lay supine in the scanner with their heads fixed using foam cushions to minimize head motion and they were instructed to remain as still as possible during data acquisition. Scanning started with an anatomical scan, followed by a motor execution *functional localizer* run (LOC). The LOC run served to identify functionally relevant voxels and to calculate individual percent signal change (PSC_LOC_) to scale the visual feedback. The motor execution LOC run (272 vol) consisted of four blocks of bilateral finger opposition, flanked by five rest blocks (each block consisted of 16 vol and lasted for 24.8 s). Once target regions (M1 and SMA) were identified, the session proceeded with five *neurofeedback* runs (NF) with one ROI, followed by five NF runs with the other ROI, using a counterbalanced order across participants who were blinded to the order and target region they received feedback from.

If feedback information was provided from the SMA, we labelled it an *active* condition for SMA and a *passive* condition for M1, and vice versa. NF runs consisted of 180 vol each, and contained two repetitions of two block types, a *low* and *high* neurofeedback level, which were flanked by rest blocks (each block consisted of 20 vol and lasted for 31 s). Thus, the study had a factorial design, in which for each ROI there were two conditions (*active* and *passive*), each with two levels (*high* and *low*). Participants were instructed to use kinesthetic motor imagery involving both hands during fMRI-NF and to avoid any movement or muscle contractions.

#### 2.1.9 Target region selection and feedback normalization

An incremental GLM was used for online analysis of both LOC and NF runs (Version 3.0, Brain innovation, Maastricht, The Netherlands); the model included a single task predictor for both LOC and NF. For LOC, a linear drift term was also included.

The selection of voxels for the target regions was guided by use of t-statistic maps (with a variable t-threshold, but at least t = 5.0), as well as anatomical landmarks. For M1, we aimed to restrict the ROI to the hand knob area (Yousry et al., 1997). For the SMA ROI, we aimed to target the proper SMA, and avoid selecting voxels in the pre-SMA. Local gyri and sulci have been shown to be rather variable between subjects and be less reliable, than for instance, white matter tracts (Behrens et al., 2006). To identify proper SMA, we thus used as an anterior border, the vertical line traversing the anterior commissure (VCA), which is perpendicular to the AC/PC plane (Picard and Strick, 2001; Zilles et al., 1996). Voxels were selected in both hemispheres within 3 slices and with a similar number of voxels per hemisphere and per target region (in total 36 voxels with 18 voxels per hemisphere for M1 and SMA; 6 voxels per slice).

Based on selected target regions, the percent signal change (PSC) during the motor execution localizer was calculated for both regions (M1 and SMA) separately. This allowed us to calibrate the feedback presentation based on PSC values from the respective target region and thereby to account for differences in the hemodynamic response (Obata et al., 2004).

### 2.2 Real-time fMRI analysis, neurofeedback calculation and presentation

For feedback presentation during NF runs, the mean raw signal value was extracted from the ROI at the analysis computer and transmitted to a stimulation computer at every TR via a direct TCP/IP network connection to minimize delays in data transmission. The percentage signal change (PSC_NF_) was provided as a feedback signal and was computed based on equation (1),

(1) $$PSC_{NF}=\frac{\left(val-baseline\right)*100}{baseline}$$

where *val* is the mean of the last three consecutive mean ROI raw signal values, and *baseline* is the median ROI signal value during the second half (i.e. last 10 TRs) of the preceding rest period. PSC_NF_ were then normalized by PSC_LOC_ to map it on to the (15) segments of the thermometer display such that every segment represents 10% of the PSC_LOC_. Values below 0 were rounded up to 0, values above 15 were rounded down to 15. The calculation was carried out in an in house written Python script (Python 2.7.10). The Open Source Python library Expyriment was used for online feedback presentation (Krause and Lindemann, 2014). The visual feedback display consisted of a thermometer with 15 segments and indications for either rest (i.e. red arrows at the bottom) or the target levels (Fig. 1C). Target levels were defined by 50% (*low*) and 100% (*high*) of the PSC_LOC_ and indicated by green arrows. Both the *low* and *high*-level conditions were repeated twice per run and were interleaved by rest periods. During rest periods, no feedback was presented, and the thermometer remained empty. The order of the condition (*low* and *high* target level) was counterbalanced across runs and subjects.

## 3 Analysis

### 3.1 Offline fMRI analysis

All subsequent results are based on an offline analysis of raw data that was performed in AFNI (version 16.2.18). Raw data were motion corrected and spatially smoothed (4 mm FWHM) with pre-processing parameters identical to the online analysis. Mean ROI time series were then extracted for all runs and subsequent analysis was restricted to these. Since ROI mean time series were extracted from both ROIs for each run, for each ROI there exists a condition in which it was the “active” target for NF training (*active*), as well as a condition in which NF training was performed on the other ROI (*passive*). Thus, for each ROI, the *active* condition contains the response to motor imagery when feedback was provided from that region, and the *passive* condition contains the response to motor imagery when feedback was provided from the other region. Therefore, the *passive* condition served as a form of internal (offline) control condition for a given ROI, because it reflects the response to motor imagery in that ROI when NF training is based on the feedback signal from the other ROI.

For LOC and NF runs a GLM analysis of the data was performed. For LOC runs, the model included a predictor for activation blocks and a linear drift term. For NF runs a predictor that modelled the NF response across both levels was included, as well as a parametric predictor that modelled the difference between levels. No linear drift term was included, but separate pre-onset baseline period predictors were included to match the online analysis baseline period (i.e. 10 vol preceding stimulus onset). Thus, the offline analysis replicated the online analysis, except for the addition of pre-whitening to account for temporal auto-correlation of the BOLD signal (Woolrich et al., 2001).

Offline PSC values were defined similarly to the online analysis, as a ratio between task response and baseline estimation. However, in the offline analysis the LOC baseline period was simply represented by the intercept term in the model (as is the norm for standard analysis). For NF runs, PSC values were calculated for *low* and *high* levels separately, and the baseline period was represented by the intercept term plus the pre-onset baseline period that was used in the online analysis. In this way, the offline NF PSC values are representative of the average online NF PSC values.

### 3.2 Statistical analysis

The main hypotheses were tested at the group level through the use of ANOVA models. For each subject, median PSC values were calculated across all runs for each ROI in both *active* and *passive* conditions, and for the *low* and *high* target level separately. For each ROI data were fit to a 2x2 ANOVA model (target level x feedback), with target level having *low* and *high* levels and feedback with *active* and *passive* conditions. Physiological traces (P_ET_ CO_2_ and HR) were convolved with respective hemodynamic response functions, and correlated with the task predictor (Pearson's r). Obtained correlation coefficients were Fisher-z-transformed for both measurements and ROIs, averaged across runs, and submitted to one-sample t-tests.

All frequentist t-tests were carried out one-sided, unless stated otherwise. Bayesian paired t-tests were conducted using JASP (version 0.8.6)(Team, 2018) using informed half-normal priors. Priors that were used to test for M1 activity were scaled by 75% of the group M1 PSC value as measured during the functional localizer and priors to test for differences between *low* and *high* target level of SMA activity were scaled by 50% of the group SMA PSC as measured during the functional localizer, which reflects the scaling of target levels during online feedback (for more details on used prior distributions, see Results section).

## 4 Results

### 4.1 Motor imagery questionnaire

Motor imagery questionnaire data were collected from 14 of 17 participants. Data suggested that participants reported competence in imagining movements visually (5.5 ± 0.2 points [Mean ± SEM]) and kinesthetically (5.4 ± 0.2 points), as indicated by the average ratings. The sum scores for visual (38.4 ± 1.7; range 24–48) and kinesthetic (35 ± 1.6; range 28–44) motor imagery were also comparable. During administration of the questionnaires, all subjects reported that they understood the difference between the two forms of motor imagery and provided examples for illustration. Hence, we concluded that participants in our sample were able to engage in kinesthetic (upper-limb) motor imagery.

### 4.2 Percent signal changes in target ROIs

**Fig. 2:**
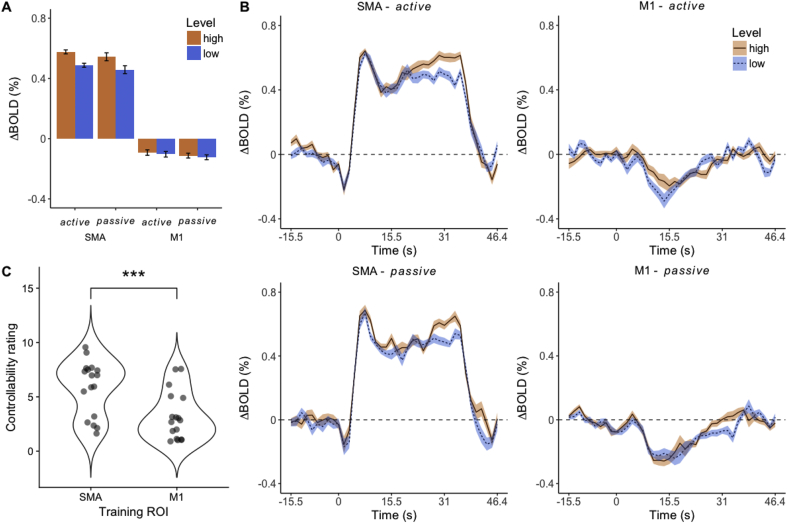
A) Bar plots for BOLD percent signal changes (PSC) in the target ROIs during the active and passive conditions (Mean ± within subject standard error). B). Event related BOLD activity in target ROIs during the active and passive conditions. Shown are group mean values and within-subject standard error around the mean (shaded). C) Voilin plot showing the Controllability ratings for SMA and M1, *** significant difference p < 0.001.

Group mean PSC values during the motor execution (finger opposition) localizer task were 1.48 ± 0.12 [SEM] for M1, and 1.10 ± 0.08 for SMA, respectively. Fig. 2A shows the mean BOLD responses across subjects and runs for each ROI in both the *active* and *passive* neurofeedback conditions. In Fig. 2B, a robust positive BOLD response during the task period can be seen in the SMA for both feedback conditions, whereas the M1 shows a clear negative response. These event related time courses indicate that the SMA showed sustained activation during the task period, while M1 was deactivated irrespective of whether feedback was provided from M1 or the SMA. M1 deactivation was confirmed by a *t*-test based on participants' M1 PSC values averaged across the *low* and *high* feedback level during the task period (M1 *active:* t_16_ = −2.196, p = 0.022; Cohen's d = −0.533; M1 *passive*: t_16_ = −3.552, p = 0.002; Cohen's d = −0.862). This was supported by Bayesian t-tests (N(0, 1.11); M1 *active*: BF_-0_ = 3.496; M1 *passive*: BF_-0_ = 44.241).

Next, we tested for differences in SMA activation during the low and high neurofeedback condition. SMA PSCs values during SMA (*active*) and M1 (*passive*) training were submitted to a 2 (*low* and *high* target level) x 2(*active* and *passive* condition) repeated measures ANOVA. We found an effect of level (F_1,16_ = 7.334, p = 0.016, **ω**^**2**^ = 0.26). However, there was no effect for feedback condition (F_1,16_ = 0.231, p = 0.637, ω^2^ = 0.0), nor an interaction between feedback condition and level (F_1,16_ = 0.005, p = 0.947, ω^2^ = 0.0). We thus only followed up level effects for SMA PSCs pooling across *active* and *passive* SMA PSCs and found a significant difference of moderate strength (Mean difference: 0.087 ± 0.029 PSC, t_16_ = 3.006, p_bonf_ = 0.005, Cohen's d = 0.729). Likewise, a Bayesian *t*-test suggested strong evidence for a greater PSC during the *high* compared to the *low* level condition (N(0, 0.55); BF_+0_ = 14.61). However, we note that this effect was likely mainly driven by PSCs from the *active* SMA condition, as suggested by a descriptive comparisons of effect sizes (*active*: Cohen's d = 0.818, 95% Confidence Interval [0.257 to 1.361]; *passive*: Cohen's d = 0.393, 95% CI [-0.107 to 0.882]). Lastly, to test for an effect of time in level separation, difference scores (*high* vs. *low* level) for each *active* run were submitted to a repeated measures ANOVA. No effect of time was found for SMA (F_4,52_ = 1.199, p = 0.322, ω^2^ = 0.014) or M1 F_4,64_ = 1.125, p = 0.354, ω^2^ = 0.007) fMRI-NF training. Taken together, we found evidence for M1 deactivation irrespective of the feedback condition. Further, SMA PSC data suggested a main effect of the motor imagery level, which seemed largely driven by the *active* SMA neurofeedback condition.

### 4.3 Physiological measurements

As with any BOLD fMRI study, magnitude changes can be confounded by non-neural sources of variance, such as motion and physiological noise. Furthermore, physiological processes may be modulated by underlying mechanisms that also respond to the cognitive demands of a task, such as arousal or concentration, e.g. increased heart-rate during arousal or subconscious changes in breathing during increased mental activity. Specifically, partial pressure end-tidal carbon dioxide (P_ET_ CO_2_) acts as a strong vasodilator, and heart-rate (HR) impacts the BOLD signal (Murphy et al., 2013). To account for potential physiological confounds (Thibault et al., 2018), we collected P_ET_ CO_2_ and HR traces and correlated these with the task predictor. In general, small negative relationships were found, except for HR in M1 (see Table 1), although these were not significant after multiple comparison correction. Also, the Bayes Factor remained inconclusive for all measurements and ROIs, suggesting that correlations between physiological confounds and the task were rather negligible.

**Table 1:** Correlations between physiological parameters and motor imagery task predictor for heart-rate (HR) and pressure end-tidal carbon dioxide (P_ET_ CO_2_) for both training ROIs. Descriptive and inferential statistics (one-sample *t*-test) of z-transformed correlation coefficients. Shown are Mean and Standard Error of Mean (SEM) values and p-values before and after (FDR) correction. 95% CI = 95% Confidence Interval, BF = Bayes Factor.

	z (Mean ± SEM)	t	df	p	p_FDR_	Cohen's d [95% CI]	BF
HR SMA	−0.06 ± 0.04	−1.359	9	0.207	0.277	−0.43 [-1.07 to 0.23]	1.022
C02 SMA	−0.08 ± 0.03	−2.351	12	0.037	0.148	−0.65 [-1.24 to −0.04]	2.915
HR M1	0.02 ± 0.05	0.336	10	0.744	0.774	0.10 [-0.49 to 0.69]	0.540
C02 M1	−0.07 ± 0.04	−1.920	13	0.077	0.154	−0.51 [-1.06 to 0.05]	1.765

### 4.4 Post-training questionnaire

Lastly, we tested whether differences between ROIs (SMA and M1) in up-regulation were reflected in reported experiences. Hence, we first assessed how participants rated the controllability of the feedback during M1 and SMA training. Fig. 2C shows participants rated controllability higher for the SMA (t_16_ = 4.28, p < 0.001, 5.8 ± 0.6 vs. 3.2 ± 0.5, Cohen's d = 1.0), for which the Bayes Factor suggested strong evidence (N(0, 0.5); BF_10_ = 53.5). The SMA was also rated as the better region with regards to the perceived ability to fill up the thermometer bars (t_9_ = 6.89, p < 0.001, 3.5 ± 0.7, vs. 8.7 ± 0.4, Cohen's d = 2.2), and to maintain bars at target levels (t_9_ = 5.16, p < 0.001, 5.2 ± 0.6, vs. 8.6 ± 0.4, Cohen's d = 1.6). For both effects Bayesian t-tests suggested strong evidence (N(0, 0.5); BF_10_ = 52.08 and BF_10_ = 24.29, respectively). Taken together, ratings of controllability reflect that participants' perception of control of the feedback during SMA neurofeedback training and their inability to self-regulate M1.

## 5 Discussion

### 5.1 Main findings

Our main finding is that kinesthetic motor imagery did not elicit a positive BOLD response in the M1 region of motor cortex, irrespective of whether of feedback information was provided. In fact, we observed a significant negative BOLD signal change for both conditions. This result has considerable implications for motor imagery based fMRI-NF in particular because it suggests that upregulation of the M1 BOLD signal is precluded by a negative activation during motor imagery. The inability of participants to upregulate M1 BOLD is also reflected in subjective ratings of controllability, which were low. This observation has wider implications for any fMRI-NF paradigms that employ specific cognitive strategies, because it highlights the critical importance of selecting regulation objectives that are achievable with a given choice of target region and cognitive strategy.

A second target area, the SMA, was also trained in separate runs, and consistent with previous reports (Subramanian et al., 2011, 2016), we found that participants did show activation during motor imagery. However, this in itself is not sufficient to demonstrate volitional control, because it is established that the SMA is activated during motor imagery in general, without feedback being necessary. The proposed benefit of neurofeedback as a therapeutic tool is based on the rationale that participants can self-regulate their own brain activity, and that this volitional control can be used to modulate brain states that can lead to a clinical benefit. This requires demonstrating genuine neurofeedback effects that extend beyond mere motor imagery training effects, and mere psychosocial effects (Thibault et al., 2018). The strength of the present study lies in the use of a graded NF paradigm, because the targeting of discrete BOLD response magnitudes presumably requires some degree of self-regulation that would benefit from a feedback loop. We found a main effect of feedback level on SMA PSC values, which demonstrated significantly higher BOLD responses during in *high* compared to *low* levels, but no interaction between target level and feedback condition. Moreover, we addressed the concern of potential physiological confounding factors (Thibault et al., 2018) and showed that P_ET_ CO_2_ and HR did not correlate significantly, and numerically only to a small degree with self-regulation blocks (Table 1).

### 5.2 M1 deactivation

The role of M1 in motor imagery is likely complex and context dependent and subject of an active debate (for a review see (Hetu et al., 2013; Munzert et al., 2009);). For instance, an Activation Likelihood Estimation (ALE) meta-analysis based on 73 published fMRI motor imagery studies found no conclusive results for M1 activation and noted that only 22 out of 122 neuroimaging studies (fMRI and PET) reported M1 activation (Hetu et al., 2013). The authors concluded that imprecise motor imagery instructions may explain some of the inconsistency of M1 findings reported in the motor imagery literature, and it further been suggested that only kinesthetic motor imagery may elicit M1 activation (Blefari et al., 2015; Solodkin et al., 2004). In this study, we have more rigorously controlled for both these factors by providing clear instructions and instructing participants to exclusively use kinesthetic motor imagery of actions that involved both hands. The use of SMA as a separate ROI, and the demonstration of a robust positive BOLD response in this area, also served as a control condition, and strongly negate any notion that participants were simply unable to engage in motor imagery. We have demonstrated that M1 (hand knob) could not be activated despite reinforcing feedback and conclude that it likely not involved in motor imagery, which would explain previous unsuccessful M1 fMRI-NF attempts (Berman et al., 2012; Blefari et al., 2015; Chiew et al., 2012). However, we also note that these reported attempts as well as the present study comprised a single session. Hence, it remains possible that participants can activate M1 and gain volitional self-regulation with more training experience, for instance after multiple sessions of M1 up-regulation fMRI-NF.

One potential confounding factor of motor imagery paradigms is movements or subtle muscle contractions, which could lead to spurious activations within regions of interest (Thibault et al., 2018). The few studies that have reported M1 activation during motor imagery mostly did not control (Lotze et al., 1999; Perronnet et al., 2017; Sharma et al., 2008) for overt movements and muscle contractions, for which Electromyography (EMG) recordings are required. Likewise, this study did not record EMG to rule out muscle activity and this limitation should be addressed in future studies. However, the within subject design and comparison of separate ROIs limits the likelihood that participant motion (either voluntary or involuntary) contributes in any systematic way. Importantly, our main finding of M1 deactivation during motor imagery periods strongly suggests that the presented results were likely not confounded by participants’ overt movement production, because these would result in a positive BOLD response in the M1 target region whose voxels were selected based on a motor execution localizer.

### 5.3 Neural origin of M1 deactivation

One potential mechanistic explanation for the observed M1 deactivation during motor imagery could relate to the suppression of overt movements. Invasive electrophysiological stimulation studies have shown that M1 can have suppressive effects on muscular activity in motor control (Ebbesen and Brecht, 2017). In our motor imagery paradigm participants were required to avoid muscular activity, and it is plausible that inhibition of movement is realized in the primary motor cortex, which acts as the final station where motor commands converge before being executed. Previous fMRI motor imagery studies have reported inhibitory projections from the SMA to M1 in healthy participants (Kasess et al., 2008; Solodkin et al., 2004) and stroke survivors (Bajaj et al., 2015b), which could explain the M1 BOLD deactivation reported here. Moreover, one motor imagery study conducted in stroke survivors reported an inverse relation between (self-reported) motor imagery and activity of M1, indicating that individuals with higher motor imagery capacity showed more suppression of M1 activation (Confalonieri et al., 2012).

Electrophysiological studies suggest that M1 is involved in motor imagery and can provide further insight into the neural origin of M1 BOLD deactivation. For instance, one magnetencephalography study reported activity in the hand area of M1 during motor imagery (Schnitzler et al., 1997). More direct evidence for the involvement of M1 in motor imagery stems from a electrocorticography (ECoG) study that reported decrease of power (Miller et al., 2010) within lower frequencies reported for motor imagery (Pfurtscheller and Aranibar, 1979; Pfurtscheller and Neuper, 1997), including the Mu-rhythm (8–13 Hz). Similar findings have been reported in BCI that could demonstrate training effects in motor imagery paradigms (for a review see (Cervera et al., 2017);). A power decrease of the Mu-rhythm during unilateral hand motor imagery has further been associated with increased spinal motor neuron excitability (Takemi et al., 2015), as well as increased excitability of the corticospinal tract and intracortical GABAergic inhibition (Takemi et al., 2013). Simultaneous EEG-fMRI recordings have linked the Mu-rhythm to a decrease in BOLD deactivation (Mullinger et al., 2014). Taken together, some electrophysiological correlates of motor imagery anti-correlate with BOLD and may hence partly explain the presented M1 findings.

### 5.4 Graded neurofeedback

The second aim of this study was to test if participants could exert volitional control over the BOLD signal, in either SMA or M1 during kinesthetic motor imagery, to target two discrete BOLD signal change magnitudes. We found a significant main effect of target level for SMA PSCs in general, but no interaction between target level and feedback condition (*active* vs *passive*). Thus, although there was a significant difference between SMA PSC in *high* vs *low* target level conditions, this effect was not significantly modulated by the feedback condition. However, a comparison of effect sizes suggested that the level effect was mainly driven by the *active* condition. As such it appears that graded fMRI-NF does aid participants in achieving discrete levels, but the specific neurofeedback effect of volitional control of BOLD signals was relatively small compared with the general motor imagery effect. More neurofeedback training sessions may be required to demonstrate that participants benefit from feedback by utilizing this information to achieve self-regulation of discrete BOLD target levels.

We note that a previous study that investigated (3-level) graded fMRI-NF for different mental imagery tasks found superior self-regulation effects within a single training session when comparing fMRI-NF to a pure mental imagery session (Sorger et al., 2016). However, one main difference between the study designs lies in the selection of target regions: whereas the present study used a motor execution task, Sorger et al. (2016) used a mental imagery task to identify target regions and maximum PSC to scale the feedback, and thus possibly targeted voxels that are more specifically involved in the respective mental imagery task. Additionally, LOC PSC values were on average larger than in our study. Although it remains speculative, participants may in consequence have had more dynamic range to exploit of the BOLD signal. This notion seems supported by a recent BCI study that used a graded feedback approach and found a positive relationship between accuracy of self-regulation and maximal localizer PSC values that were used to scale the individual feedback (Krause et al., 2017).

### 5.5 Implications for motor neurofeedback

We have provided new insights into neural correlates of (supervised) motor imagery, which can inform future attempts of translating fMRI-NF to clinical applications. In particular for stroke rehabilitation, fMRI-NF may provide new means to enhance neuroplasticity (Wang et al., 2017). One possible target region could be M1, which has been intensively studied in brain stimulation based stroke rehabilitation (McDonnell and Stinear, 2017). Considering the presented findings, however, M1 is likely a suboptimal target region for motor imagery based fMRI-NF upregulation training, although it remains possible that M1 activation can be achieved with longer training. We note that our results are largely in line with findings from Hanakawa and colleagues who had compared motor networks during motor planning, motor imagery and motor execution (Hanakawa, 2016). The authors had classified brain areas as “movement-predominant”, which included the M1 and SMA, and as “imagery-predominant”, which included the SMA but not M1. Further, a direct comparison between tasks suggested that associated motor networks largely overlapped, but also that voxels that were more responsive to motor execution were located more anteriorly within the SMA and the precentral gyrus. In contrast, voxels that were more responsive to motor imagery were located more posterior within these areas. This observation may be of relevance for the interpretation of the presented findings, because training voxels were identified using a motor execution localizer. To achieve optimal voxel selection future studies may benefit from employing a motor imagery based localizer scan. One alternative approach to test if M1 activation can be learned could be realized in a design that does not use explicit motor imagery strategies whilst controlling for movements. Another set of techniques that could yield control over M1 is multivariate fMRI-NF, given that in previous studies, different imagined actions could be decoded from M1 (Park et al., 2015; Pilgramm et al., 2016; Zabicki et al., 2017).

We note that the presented findings are confined to young healthy participants and may not generalize to patient populations. For instance, stroke survivors show altered motor networks (Rehme et al., 2011, Rehme and Grefkes, 2013), implying that motor areas in the contralesional hemisphere can partly compensate for motor impairment. Furthermore, inter- and intrahemispheric network activity alters over time, hence optimal target regions may also depend on the time after stroke, in addition to other factors such as the lesion location (e.g. hemisphere), and involvement of the corticospinal tract (Stinear et al., 2007). For instance, individuals with right-hemispheric stroke seem to show more frequently impairment of motor imagery than individuals with left-hemispheric stroke (Kemlin et al., 2016). M1 remains a promising target region for motor execution-based fMRI-NF training for individuals who possess sufficient residual motor function. The literature on motor execution-based fMRI-NF training is currently limited to one study in healthy participants that reported training success for the M1 hand knob area (Neyedli et al., 2017).

With regards to the SMA as a target region, besides its role in motor control, it is involved in vigilance more generally (Cunnington et al., 2002; Hinds et al., 2013), as well as attention to timing (Coull et al., 2004; Macar et al., 2006). Both cognitive processes are likely recruited during the attempt to self-regulate brain activity, for instance participants likely monitor their self-regulation performance and attend to the temporal delay of the visual feedback display. Although we could control for general mental imagery effects by comparing PSCs obtained from the SMA *active* and the SMA *passive* condition, such cognitive processed that involve the SMA may have interfered with participants attempt to increase and decrease SMA activity while targeting discrete BOLD levels. It thus remains to be tested whether self-regulation success in motor imagery based graded fMRI-NF could be increased by targeting other premotor areas (e.g. vPMC), using different a form (e.g. intermittent (Hellrung et al., 2018);) or modality (e.g. auditory) of feedback presentation, or employing a different feedback type (e.g. connectivity based feedback (Liew et al., 2016);).

## 6 Conclusion

In this study we have demonstrated that M1 shows a robust negative BOLD response during kinesthetic motor imagery in the context of NF. This finding may explain previous unsuccessful attempts of M1 fMRI-NF, suggesting that it represents a suboptimal target region for upregulation paradigms in which participants use explicit kinesthetic motor imagery. Conversely, premotor cortical regions such as SMA are more suitable target regions because of their more active role in motor imagery in general. However, the robust reactivity of premotor areas like SMA to different forms of motor imagery also presents a confound in NF studies that aim to develop volitional self-regulation of neuronal activation, as additional evidence beyond simple positive BOLD responses is required to demonstrate volitional self-regulation.
